# Book Review: Cybercognition: Brain, Behaviour and the Digital World

**DOI:** 10.3389/fpsyg.2018.01069

**Published:** 2018-06-26

**Authors:** Gavan Lintern

**Affiliations:** Accident Research Centre, Monash University, Melbourne, VIC, Australia

**Keywords:** cyberspace, digital cognition, digital distraction, multitasking, information search, online credibility

The Internet and the devices through which we access it have changed the way we live, work, and interact with others. In *Cybercognition*, Hadlington explores two questions; in accessing the virtual world of the Internet, are we different cognitively than when we operate in the physical world, and do our interactions with this virtual world change us cognitively. The title, *Cybercognition*, refers to our cognitive engagement with that online, virtual world we might term cyberspace.

Hadlington reflects on a broad sampling of cognitive research to explore questions such as; does experience with video games and computer-based exercises improve our cognitive functions, what is multitasking and do we get better at it, how are the frequent interruptions we get from our digital devices affecting the way we do other things, how are digital technologies affecting education, and how might we assess the credibility of information we find on the Internet. In this review, I will touch on just a few issues covered in the book.

Cyberspace is a source of interruption and distraction. Although both have always been with us, their presence in our lives is accentuated by the ubiquity of digital technologies. Does it matter? The evidence points to both being a problem. Interruptions (emails, text messages, digital alerts) not only divert us from what we are doing for the time of the interruption, but negatively impact the quality of our work. Furthermore, we often do not return to complete what we were doing when we were interrupted.

As digital technologies have proliferated, multitasking has emerged as a socially significant construct. The popular notion of a supertasker who can engage in several complex activities at once is coupled with a belief that the more we multitask, the better we become at it. The evidence suggests that these beliefs are wrong. Indeed, we do not multi-task in the sense that we attend simultaneously to more than one cognitively demanding activity. Rather, we switch between activities, interleaving in a way that has us frequently suspending and resuming tasks, focusing on one at the expense of the others.

Problematically, there are cognitive costs associated with resuming a suspended task, and if any one of these tasks is safety-critical, so-called multitasking can increase the risk of accident. Some of us think we are immune to the danger, but research suggests that those of us who think we are good at multitasking are less able to do it than those of us who choose to avoid it. There has been much written on the risks associated with mobile phone use while driving. This was once viewed as an issue of physical interference from handling the device, which could be resolved by use of hands-free devices, but there is mounting evidence that even hands-free telephone conversations increase accident risk. This would seem to be an issue of cognitive interference rather than of physical interference.

Some decades ago, a colleague and I commented on claims that experience with action video-game skills could develop capabilities that could generalize to control of complex, high-performance vehicles (Wightman and Lintern, 1985). At the time, there was little evidence to assess that belief. Hadlington has reviewed evidence that has accumulated more recently. Nothing much has changed. There is still enthusiasm for the potential but the evidence for the affirmative remains thin and controversial. The situation in relation to brain training is similar. Brain training, more appropriately designated as cognitive training, is based on the hypothesis that intensive practice of certain cognitive skills, as embedded in specially tailored computer exercises, will enhance cognitive performance in normal everyday life. There are some positive results, but they are small and isolated. Furthermore, failures to replicate, combined with critiques of method, cast doubt on the robustness and generalizability of the positive results.

Cyberspace is a world in which the old rules for assessing trustworthiness of information have been eroded. Anyone can publish anything, which then becomes accessible to anyone anywhere who has a digital device. Those who wish to promote their views can bypass the traditional filters imposed by editors, reviewers, and the like. Any tendency we might have to trust the written word is challenged. Nor can we rely on the physical signs of deception that can be informative face-to-face. There are heuristics we can use, such as comparing information from multiple online sources, but those who wish to persuade us against our better judgement can present information in ways that hinder our capacity for sensible judgement by obscuring the traditional, physical-media boundaries between fact, opinion, and persuasion.

What to make of Hadlington's exploration of cybercognition? There are warning signs but also opportunities. Some have become addicted to their digital technologies, but those same technologies help us share information and interact socially and professionally to an extent not previously possible. Smart phones may compromise learning by acting as a source of distraction, but digital technologies, if used in a targeted and contextually specific manner, may support education in new and innovative ways. The challenge, it would seem, is to build on the opportunities while we find ways to ameliorate the problems.

To reflect on the first of Hadlington's questions, are we doing anything different cognitively in cyberspace vs. the physical world? The answer is mixed. With interruptions and multi-tasking, it would seem so. We are at least in a more extreme environment. In contrast, we are in something of a middle ground with information search, where we appear to be using physical-world strategies in combination with cyberspace-specific strategies. And then, in establishing credibility, we appear to be facing a new situation where we have been forced to develop new strategies.

In reflecting on the second of Hadlington's questions, are our interactions with this virtual world changing us cognitively, we find a body of research that is so fragmented and questionable that it is hard to draw any conclusion. One of the primary messages from Hadlington is that we need more research in this area. I generally agree with this conclusion, but in a cyberspace-dominated world where trust is eroding, it needs to be definitive research. To my mind, if cyberspace really is changing us cognitively, it should not be too hard to demonstrate that empirically. So far, we have failed.

In planning this book, Hadlington has converged on a set of topics at the forefront of popular discussions surrounding the benefits and evils of cyberspace. However, potential readers might be warned. This is not a Malcolm-Gladwell quasi-science infotainment. Rather than selecting research to support a good story, Hadlington offers a balanced sampling, one that more truthfully represents what is known in this area. This book is not as entertaining as a Gladwell narrative, but it is interesting and readable, and it offers a balanced perspective on cyberspace issues at the forefront of popular consciousness. If you are looking for information and credibility rather than entertainment, this is a good place to start.

## Author contributions

The author confirms being the sole contributor of this work and approved it for publication.

### Conflict of interest statement

The author declares that the research was conducted in the absence of any commercial or financial relationships that could be construed as a potential conflict of interest.
